# Successful recanalization post endovascular therapy is associated with a decreased risk of intracranial haemorrhage: a retrospective study

**DOI:** 10.1186/s12883-015-0442-x

**Published:** 2015-10-07

**Authors:** David T. Wang, Leonid Churilov, Richard Dowling, Peter Mitchell, Bernard Yan

**Affiliations:** Department of Radiology, Royal Melbourne Hospital, University of Melbourne, Parkville, VIC Australia; Florey Institute of Neuroscience and Mental Health, Melbourne, Australia; RMIT University, Melbourne, Australia; Melbourne Brain Centre, Royal Melbourne Hospital, University of Melbourne, 300 Grattan St, Parkville, Victoria 3050 Australia

**Keywords:** Stroke, Intra-arterial therapy, Intracranial haemorrhage

## Abstract

**Background:**

The risks of intracranial haemorrhage (ICH) post intra-arterial therapy (IAT) for stroke are not well understood. We aimed to study the influence of recanalization status post IAT for anterior circulation stroke and posterior circulation stroke on ICH development.

**Methods:**

Retrospective analysis of 193 patients in a prospectively collected database of IAT stroke patients was performed. Successful recanalization was defined as a Thrombolysis in Cerebral Infarction Score of 2b or 3 and symptomatic ICH (SICH) as parenchymal hematoma type 2 (PH2) with neurological deterioration. The association between the recanalization status and ICH/SICH was investigated using logistic regression models adjusted for baseline characteristics selected by univariate analyses.

**Results:**

One hundred and thirty-six patients had successful recanalization post procedure, 41 patients developed ICH and 10 patients SICH. There was a statistically significant baseline imbalance between the groups with and without successful recanalization on gender, baseline National Institute of Health Stroke Scale (NIHSS) score, the use of intravenous tPA and intra-arterial urokinase (*p* <0.05). Logistic regression analysis adjusted for the above variables and the time to digital subtraction angiography demonstrated a statistically significant association between successful recanalization and ICH (odds ratio 0.42; 95 % CI 0.19, 0.95; *p* = 0.04).

**Conclusion:**

Successful recanalization post endovascular therapy is statistically significantly and negatively associated with ICH.

## Background

Intracranial haemorrhage (ICH) is a significant complication of both intravenous tissue plasminogen activator (IV tPA) and intra-arterial therapy (IAT). Parenchymal haemorrhage is associated with poor clinical outcomes, with increased rates of deterioration after 24 h, disability and death after 3 months post stroke [1]. The incidence of ICH is similar between IAT and IV tPA, reaching up to 43 % in some studies [2]. To date, IV tPA or IV tPA with IAT are the only proven therapy for acute ischaemic stroke [3]. However, IV tPA by itself is associated with low rates of recanalization (38 % for internal carotid artery, 44 % for M1, 44 % for single M2 occlusion), compared to 65 %, 81 % and 70 %, respectively for IAT [4]. Given the uncertain efficacy of IAT, there is emerging interest in the identification of risk factors for ICH post intervention for safety purposes.

Animal studies showed increased incidence of haemorrhagic transformation post IV tPA with increasing duration of arterial occlusion [5–7]. In human studies, delayed recanalization was also associated with haemorrhagic transformation [8, 9]. Disruption to the blood brain barrier was the presumed culprit mechanism for the development of haemorrhagic transformation. Blood brain barrier failure was thought to progress in steps, from reactive hyperaemia to hypoperfusion, resulting in increased paracellular permeability. The estimated time to blood brain barrier disruption was posited at approximately 3.8 h [10].

The associative factors of post-tPA related ICH have been well studied [11] and included high National Institutes of Health Stroke Scale (NIHSS) score, brain oedema, hyperglycaemia, mass effect and early ischaemic changes on neuroimaging [12]. On the other hand, there is less data on the predictors of intracranial haemorrhage post IAT.

The aim of our study was to investigate the influence of recanalization success on the incidence of ICH in patients treated with IA therapy for acute ischaemic stroke. We hypothesised that successful recanalization in stroke patients is associated with decreased risk of ICH.

## Methods

This research was approved by the Human Research Ethics Committee of Royal Melbourne Hospital, in compliance with the Helskinki Declaration, and has waived consent for this research.

A retrospective analysis of a prospectively collected database of all patients from a single centre, who underwent intra-arterial therapy between October 2007 and November 2013, was performed. The selection for the patients for intra-arterial therapy included patients with CT or cerebral digital subtraction angiogram (DSA) evidence of: (1) M1, M2 or internal carotid artery occlusion within 4.5 h of stroke symptoms; or (2) occlusion in the basilar or vertebral arteries within 24 h of symptoms. There were no age restrictions. Patients with a pre-existing modified Rankin Scale (mRS) >3, plain CT or CT perfusion evidence of infarct core greater than one third of the MCA territory were excluded.

A range of IA treatments were available during this period including IA tPA, IA urokinase and mechanical thrombectomy (MERCI, Penumbra or Solitaire).

Data collected include patient demographics, vascular risk factors, baseline NIHSS, time from onset of symptoms to digital subtraction angiography (DSA), use of IV tPA as well as post-procedural imaging documenting recanalization as well as the presence and type of ICH on follow up CT brain. Post-treatment recanalization was classified using the Interventional Management of Stroke (IMS) II Study Thrombolysis in Cerebral Infarction (TICI) grading [13]. In keeping with previous studies, TICI grade 2a and below were deemed as unsuccessful recanalization and TICI 2b and 3 as successful recanalization [14]. ICH was sub-typed according to the European Cooperative Acute Stroke Study (ECASS) II classification [15]. SICH was defined as parenchymal hematoma type 2 (PH2) with neurological deterioration (greater than or equal to 4 point deterioration on NIHSS from baseline), as this definition has a good prediction for both mortality and outcome [16].

Cerebral DSA was performed on a biplane neurointerventional angiography unit (Axiom Artis dBC, Siemens Healthcare, Germany). CTs were performed on a 64 MDCT scanner (Somaton Sensation 64, Siemens Healthcare, Germany).

Judgement of TICI and symptomatic haemorrhage were determined by the consensus of three experienced neurointerventionalists.

### Statistical analysis

Statistical analysis was performed using Stata 13IC (StatCorp LP, Texas, USA). Univariate analysis was performed using Fischer’s exact test for categorical variables and two-sample Wilcoxon rank-sum test for ordinal and continuous variables to identify statistically significant imbalances on baseline variables between the groups with and without successful recanalization. Binary logistic regression adjusting for these baseline variables as well as for the time to DSA was then performed using recanalization status as independent variable and ICH/SICH as the dependent variable. Standard diagnostics of collinearity and assessments of model fit were performed.

## Results

A total of 193 patients underwent IAT in this time period. Median age for the cohort was 67 (IQR: 56, 75) years. Of these, 70 (36.3 %) were women, 33 (17.1 %) had diabetes, 95 (49.2 %) hypercholesterolemia, 61 (31.6 %) AF, 28 (14.5 %) ischaemic heart disease (IHD) and 22 (11.4 %) previous stroke or transient ischaemic attack (TIA). Median baseline NIHSS was 17 with 11 and 22 as the interquartile ranges. Mean time from onset of symptoms to DSA was 347.9 ± 235.6 min. The exact time of onset of symptoms was unable to be determined in 4 patients. 132 patients had anterior circulation occlusions, while 61 patients had posterior circulation occlusions.

136 patients (70.5 %) had successful recanalization post treatment (TICI grades 2b and 3).

ICH occurred in 41 patients (21.2 %) and SICH in 10 patients (5.2 %). No ICH was due to vessel perforation or rupture.

Baseline characteristics, as well as treatment method and time to treatment between the groups with successful and unsuccessful recanalization are summarised in Table 1. The baseline characteristics statistically significantly different (*p*-value <0.05) between the two groups are gender, baseline NIHSS, the use of IV tPA and IA urokinase. Median time to DSA was 287 (IQR: 238, 406) for the unsuccessful recanalization group compared to 274 (IQR: 200, 363) for the successful recanalization group (Wilcoxon-Mann–Whitney *p* = 0.14).

**Table 1:** Baseline characteristics by recanalization status

	Unsuccessful recanalization (*n* = 57)	Successful recanalization (*n* = 136)	*p*-value
Age	70 (57, 76)^a^	66 (55, 76)^a^	0.46
Gender			0.008
Male	28 (49.1 %)	95 (69.9 %)	
Female	29 (50.9 %)	70 (36.3 %)	
Type 2 Diabetes Mellitus	10 (17.5 %)	23 (16.9 %)	>0.99
Hypertension	28 (49.1 %)	67 (49.3 %)	>0.99
Hypercholesterolemia	17 (29.8 %)	40 (29.4 %)	>0.99
Atrial fibrillation	16 (28.1 %)	45 (33.1 %)	0.61
Ischaemic heart disease	7 (12.3 %)	21 (15.4 %)	0.66
Previous stroke or TIA	5 (8.8 %)	17 (12.5 %)	0.62
Baseline NIHSS	20 (14, 26)^a^	15.5 (10, 20)^a^	0.001
Time to DSA (minutes)^b^	287 (238, 406)^a^	274 (200, 363)^a^	0.14
IV tPA	8 (14.0 %)	60 (44.1 %)	0.00
IA urokinase	32 (56.1 %)	49 (36.0 %)	0.01
IA tPA	8 (14.0 %)	12 (8.8 %)	0.31
Anterior circulation occlusion	39 (68.4 %)	93 (68.4 %)	>0.99

Baseline and treatment variables, with univariate analysis. Fischer’s exact test was performed for categorical variables and two-sample Wilcoxon rank-sum test for ordinal and continuous variables. ^a^Median (1^st^ quartile, 3^rd^ quartile). ^b^Data from 4 patients unable to be calculated

Breakdown of recanalization, ICH and SICH numbers are shown in Table 2.

**Table 2:** Number of patients with ICH and SICH stratified by TICI grade

Thrombolysis in Cerebral Infarction (TICI) grade	Number of patients (*n* = 193)	ICH (*n* = 41)	SICH (*n* = 10)
Unsuccessful recanalization	57	21	6
0	25	6	2
1	10	6	2
2a	22	9	2
Successful recanalization	136	20	4
2b	39	6	2
3	97	14	2

TICI grades 0-2a inclusive are considered unsuccessful recanalization, and grades 2b and 3 as successful recanalization

There was a statistically significant inverse relationship between the recanalization status and ICH adjusted for gender, baseline NIHSS, the use of IV tPA, IA urokinase and time to DSA (adjusted OR = 0.42, 95 % CI: 0.19, 0.95, *p* = 0.038). Similar analysis did not identify a statistically significant association between the recanalization status and SICH (adjusted OR = 0.24, 95 % CI: 0.05, 1.1; *p* = 0.07). Multivariable logistic regression analyses for ICH and SICH are shown in Tables 3 and 4, respectively.

**Table 3:** Binary multivariable logistic regression analysis for ICH

Factor	Odds ratio	95 % CI	*p*-value
Successful recanalization	0.42	0.19 – 0.95	0.04
Gender	0.76	0.35 – 1.64	0.48
IV tPA	0.70	0.29 – 1.72	0.44
IA urokinase	1.52	0.71 – 3.23	0.28
Baseline NIHSS (per point)	1.04	1.001 – 1.082	0.047
Time to DSA (per minute)	1.00	0.998 – 1.002	0.98

**Table 4:** Binary multivariable logistic regression analysis for SICH

Factor	Odds ratio	95 % CI	*p*-value
Successful recanalization	0.24	0.05 – 1.10	0.066
Gender	1.72	0.39 – 7.59	0.47
IV tPA	0.83	0.14 – 4.84	0.84
IA urokinase	0.52	0.12 – 2.15	0.36
Baseline NIHSS (per point)	1.02	0.95 – 1.08	0.62
Time to DSA (per minute)	1.00	0.999 – 1.004	0.13

## Discussion

Unsuccessful recanalization at the end of IAT and higher baseline NIHSS are shown to be independent predictors of any ICH, after controlling for gender, use of IV tPA, IA urokinase and time to DSA. None of the variables identified were found to be a significant predictor of SICH.

Findings are comparable to the previous studies with IV tPA showing an association with persisting arterial occlusion and haemorrhagic transformation [8, 9].

For IAT, one study has shown the presence of diabetes mellitus and higher baseline NIHSS to be independent predictors of any ICH post IAT and the non-use of IA tPA, presence of AF and higher baseline NIHSS to the independent predictors of parenchymal haematoma [17]. Successful recanalization was not shown to be a predictor for ICH in that study. In that study, the criteria for defining recanalization was the Thrombolysis in Myocardial Infarction (TIMI) grade. The newer TICI grading is more specific for assessment of cerebral perfusion and the now widely accepted criteria for grading of successful perfusion on TICI (grades 2b and 3) is stricter than that for TIMI (grades 2 or 3). Difference in the definition for successful recanalization is the most likely reason for this discrepancy.

Our study shows that patients who had successful recanalization post IAT within 4 h for anterior circulation stroke or 12 h for posterior circulation is at a decreased risk of ICH. This is most likely because revascularisation of the brain tissue has occurred before significant failure to the blood brain barrier has occurred.

Although on the multivariable analysis, successful recanalization was not found to be significantly associated with SICH (*p*-value 0.07), this may be attributed to the small numbers of SICH (*n* = 10) and decreased statistical power to detect this effect.

It is unknown whether the association between unsuccessful cerebral perfusion and the development of ICH is cause and effect. There may be other unidentified factor or factors that make it more difficult to achieve successful recanalization, which ultimately may be the cause of ICH. On the other hand, if there is direct cause and effect, increased persistence in achieving TICI grades 2b and 3 could be pursued to decrease the risk of ICH. In the absence of definitive data, the decision for more aggressive recanalization remains a clinical one.

Baseline NIHSS is shown to be weakly associated with ICH formation but not SICH.

Limitations to this study include the fact that data is only collected from a single centre. The numbers of patients with SICH were also too small to draw confident conclusions regarding the association with SICH and recanalization. Unsuccessful recanalization was determined at the end of the endovascular procedure, with possible reopening of the artery after this point as a confounder.

## Conclusion

We showed that in our study, failure of recanalization at the end of endovascular stroke therapy is associated with ICH.
